# Operative and Practical Surgery

**Published:** 1901-03

**Authors:** 


**Operative and Practical Surgery.** By THOMAS CARWARDINE, M.S. (Lond.), F.R.C.S. Pp. xxi., 661. Bristol: John Wright & Co. 1900.

We welcome this addition to the works on operative and practical surgery chiefly on account of its real practical nature. The numerous illustrations are mostly very graphic, and show very clearly by their diagrammatic outlines the chief points they are required to delineate. Both the practitioner and the student will find the study of this volume decidedly beneficial, and at the same time pleasant reading, though in some portions the style is inclined to be "patchy."

The book is divided up into various sections, and, with the view of making the contents more useful, we should like to see some of the following alterations incorporated in the next edition :—

Psoas abscess ought to find a place in one of the sections. In reducing the dislocation at the metacarpo-phalangeal joint of the thumb we have found at times that an *open* operation without resection of the head of the metacarpal bone is required. We do not like the picture of the surgeon's foot on the pelvis of the patient with dislocated hip, and we do not think that this mode of proceeding is necessary.

In fracture of the neck of the scapula, an important sign, if present, is increased length of the limb. On reading the remarks relating to Colles's fracture, it would appear that the author retains the splints for four or five weeks, and occasionally commences passive movements somewhat earlier. If this line of treatment is adopted the patient should certainly be warned (as mentioned by the author) that many weeks may ensue before the free use of the fingers is secured. These periods of complete and partial rest should be *very* considerably shortened in our opinion.

In speaking of amputations, we quite endorse the author's views with regard to conservative surgery, as with the advances made of late years by keeping the displaced fragments of bone in apposition by screwing, nailing, and wiring, very few compound fractures ought to be amputated.

We are glad to see that so many of the lengthy and rarer operations described in other works for the radical cure of hernia have been omitted, and that the author has confined himself to describing those operations which are in more common use.

In some of the sections devoted to the surgery of the special organs too much space is devoted to uncommon diseases at the expense of those occurring more frequently, and it is doubtful whether the chapters by different authors add very largely to the value of a work of this description.

The book is clearly printed, well finished, and has a most complete index; the author is to be congratulated on the general soundness of the views expressed therein. The subject matter is well up to date, and the fund of practical information is built upon a firm basis of anatomical knowledge. We look upon this book as one of great value, and one that will repay the careful reader.

